# COVID-19: A Challenge to Physiology of Aging

**DOI:** 10.3389/fphys.2020.584248

**Published:** 2020-12-03

**Authors:** Aleksei G. Golubev

**Affiliations:** N.N. Petrov National Medical Research Center of Oncology, Saint Petersburg, Russia

**Keywords:** COVID-19, aging, theories, physiological balances, public health, anti-aging drugs, signaling pathways

## Abstract

The death toll of the current COVID-19 pandemic is strongly biased toward the elderly. COVID-19 case fatality rate (CFR) increases with age exponentially, its doubling time being about 7 years, irrespective of countries and epidemic stages. The same age-dependent mortality pattern known as the Gompertz law is featured by the total mortality and its main constituents attributed to cardiovascular, metabolic, neurological, and oncological diseases. Among patients dying of COVID-19, most have at least one of these conditions, whereas none is found in most of those who pass it successfully. Thus, gerontology is indispensable in dealing with the pandemic, which becomes a benchmark for validating the gerontological concepts and advances. The two basic alternative gerontological concepts imply that either aging results from the accumulation of stochastic damage, or is programmed. Based on these different grounds, several putative anti-aging drugs have been proposed as adjuvant means for COVID-19 prevention and/or treatment. These proposals are reviewed in the context of attributing the molecular targets of these drugs to the signaling pathways between the sensors of resource availability and the molecular mechanisms that allocate resources to storage, growth and reproduction or to self-maintenance and repair. Each of the drugs appears to reproduce only a part of the physiological responses to reduced resource availability caused by either dietary calories restriction or physical activity promotion, which are the most robust means of mitigating the adverse manifestations of aging. In the pathophysiological terms, the conditions of the endothelium, which worsen as age increases and may be significantly improved by the physical activity, is a common limiting factor for the abilities to withstand both physical stresses and challenges imposed by COVID-19. However, the current anti-epidemic measures promote sedentary indoor lifestyles, at odds with the most efficient behavioral interventions known to decrease the vulnerability to both the severe forms of COVID-19 and the prevalent aging-associated diseases. To achieve a proper balance in public health approaches to COVID-19, gerontologists should be involved in crosstalk between virologists, therapists, epidemiologists, and policy makers. The present publication suggests a conceptual background for that.

## Introduction

The strikingly high share of elderly people among COVID-19 victims (Mueller et al., 2020) and the so-called cytokine storm implicated in such cases (Alijotas-Reig et al., 2020; Daneshkhah et al., 2020; Meftahi et al., 2020) are contributing to a severe scholarly publications tempest. By the end of October 2020, the Pubmed database of biomedical publications returns more than 59,000 entries upon the query “COVID”^1^.

Amidst the resulting tumult, a fairly stable character of the pandemic has been recognized (Golubev and Sidorenko, 2020; Guilmoto, 2020; Promislow, 2020; Santesmasses et al., 2020): in different countries and at different stages of the epidemic there, COVID-19 patients at ages (*a*) ranging from 30 to 80 years feature an exponential pattern of increase in mortality rate (case fatality rate, CFR = *f*) upon increasing *a*:

$$f\,\left(a\right)=f_{0}\times e^{Y\times a}\,\mathrm{or}\,\ln f\,\left(a\right)=\ln f_{0}+Y\times a$$

In quantitative terms, the risk of COVID-19 patient death at any specific *a* may vary across countries, depending on adopted diagnostic and death attribution criteria and on the stage of COVID-19 epidemic. That is, the parameter *f*_0_ is variable. At the same time, the parameter *Y* appears strikingly stable. Its low variability makes the doubling time (DT) of CFR (DT = ln2/*Y*) to vary only from 6 to 8 years, as has been shown in the papers referenced above, whose authors have recognized this mortality pattern as consistent with the dependency of general mortality on age, which is known in gerontology as the Gompertz law or model:

$$\ln\mu \,\left(t\right)=\ln\mu_{0}+Y\times t,$$

where *μ* is mortality rate (also termed the force of mortality), *t* is the chronological age, and γ is interpreted as the rate of (demographic) aging.

The initial estimates of *Y* happened to conform to the values of γ typical for humans, i.e., 0.12 to 0.14 years^–1^ (see, e.g., Golubev A., 2019 and references therein). Such γ makes human mortality rate doubling time (MRDT) to be about 7 years. All in all, this means that, with age increasing from about 30 to 70 years, the risk of death in the symptomatic cases of COVID-19 increases by a factor of ca. 2^(75–35)/7.5^ ≈ 40, which is close to what relates to the risk of death from any cause in a general population. The process that increases the risk of dying with increasing time of living is known as aging.

It will be shown below that advances in aging research may contribute to understanding the relationships of COVID-19 pathophysiology with other salient aspects of the pandemic, including immunological, epidemiological, therapeutic, and preventive.

## Mortality Statistics as an Interface Between Gerontology and COVID-19 Research

As more data on mortality attributed to COVID-19 become available, the above quantitative estimates may require reevaluations; however, the qualitatively exponential pattern of the dependency of this aspect of mortality on age is becoming confirmed increasingly better (O’Driscoll et al., 2020). In particular, the initial estimates were based on data binned in 10-years age intervals. The above discussion will be illustrated below with data binned in 5-years intervals, as it is adopted in the Netherlands (see “Epidemiologische situatie COVID-19 in Nederland” bulletins).

**FIGURE 1:**
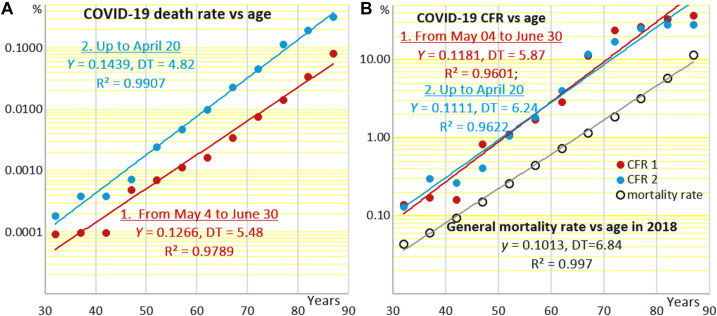
Age-specific rates of mortality attributed to COVID-19 **(A)** in the general population and **(B)** among hospitalized COVID-19 patients (case fatality rate) at different stages of the epidemic in the Netherlands. The Y-axes are semi-logarithmic. Data sources: “Epidemiologische situatie COVID-19 in Nederland” bulletins and Human Mortality Database (https://www.mortality.org/cgi-bin/hmd/country.php?cntr=NLD&level=1).

Figure 1A shows how the age-specific mortality attributed to COVID-19 depends on age in the general population. Data points are constructed by dividing the numbers of reported deaths from COVID-19 binned in 5-years age intervals, which start from 30 years, by population sizes as of 2018 in the respective age intervals, which may be found in the Human Mortality Database^2^ (HMD) and by plotting the results against the middles of these intervals. The upper series of points relates to the cumulative number of deaths that occurred in the period from the onset of the epidemic up to April 20, which includes the peak of newly diagnosed cases. The lower series relates to the period from 04 May to 30 June, when the epidemic entered the plateau phase. The different numbers of deaths related to the two periods of the epidemic (3,751 and 793, respectively), are reflected by the different positions of the respective regression lines. With that, both sets of data points fall fairly well onto straight regression lines, which are almost parallel despite all differences between the two epidemiological situations, including changes in public health approaches to dealing with COVID-19.

Figure 1B shows how COVID-19 CFR, that is the proportions of the fatal COVID-19 cases among the symptomatic patients, depend on age in the two periods of the epidemic. There is a noteworthy difference between Figures 1A and B. In Figure 1A, death rates attributed to COVID-19 are estimated against the whole populations within the respective age intervals. These death rates are not equivalent to the infection fatality rates (IFR), which must be estimated based on the numbers of people who are infected, including the asymptomatic cases. In Figure 1B, death rates are also estimated not against those infected, whose numbers are usually uncertain. Instead, they are estimated against registered symptomatic COVID-19 cases. That is why the upper death rates are less than 0.3% in Figure 1A and are as high as close to 30% in Figure 1B.

Although the two regression lines in Figure 1A are different according to the different numbers of COVID-19 death cases in the two different periods of the epidemic, they are still almost parallel. The two regression lines in Figure 1B that are based on COVID-19-attributed mortality data normalized against the registered COVID-19 cases (CFR) appear virtually identical. The estimates of CFR at 40 years derived from Figure 1B are about 0.3%. CFR estimates derived from a similar plot presented in (Golubev and Sidorenko, 2020) are 0.2% for Spain as of 23 March, 0.4 for Spain as of 11 May, and 0.75 for Sweden and Italy (13 May). These up to 3.5-fold differences in CFR are proportional to differences in *f*_0_, which thus may be quite appreciable when different countries and epidemic stages are compared. At the same time, *Y* is much more stable as reflected by mortality DT, which is about 7.5 years in Italy, Sweden and Spain and 6 years in the Netherlands.

Moreover, CFR lines in all cases here and in Golubev and Sidorenko (2020) are almost parallel to the lines that correspond to the total age-specific mortality rates as of 2018, the latest year for which mortality data are available in HMD. Correspondingly, doubling times related to CFR (DT) and to the total mortality rate in the general population (MRTD) are similar in all cases, in conformance to what has been shown earlier using less detailed data on COVID-19 epidemics in Italy, Spain and Sweden (Golubev and Sidorenko, 2020), China, South Korea, and USA (Santesmasses et al., 2020), and West Europe and North America in general (Guilmoto, 2020). DTs related to mortality attributed to COVID-19 in the general population (Figure 1A) are smaller.

Mortality rate doubling time suggests how anybody’s chances to die because of any cause during 1 year depend on age. DT suggests how the chances of COVID-19 patients (not all people infected with SARS-CoV-2, including asymptomatic subjects) to die depend on age, no time interval being specified, except for that deaths usually occur, if they do, within about three weeks after SARS-CoV-2 contraction. In both cases, the age dependencies of the chances are almost the same.

However, neither MRDT nor DT suggests how the chances to become a symptomatic (hospitalized) COVID-19 patient depend on age. This dependency is defined by the superposition of the age dependencies of the chances to: (i) be exposed to SARS-CoV-2; (ii) be infected upon exposure; and (iii) develop the recognizable symptoms upon being infected. The primary, although not exclusive, contributors to these three aspects of the overall age-dependent changes in the incidence of the symptomatic COVID-19 cases are behavioral and social, immunological, and pathophysiological factors, respectively, provided the criteria of judging COVID-19 cases as symptomatic are applied equally to all ages.

The above aspects (i) and (ii) are combined in data on age-specific SARS-CoV-2 seroprevalence. Such data collected in Spain (Pollán et al., 2020) suggest taken together, despite all local peculiarities, that, after an increase up to about 4–5% from childhood to early adulthood, there are no regular changes in SARS-Cov-2 seroprevalence throughout the rest of lifespan. This makes it almost certain that the IFR, being much lower than CFR, must still feature a similar age dependency, as has been confirmed recently (O’Driscoll et al., 2020), despite that there are several intermediate stages between registering a patient and his/her fate, including hospitalization and admission to ICU, which feature their specific age dependencies.

**FIGURE 2:**
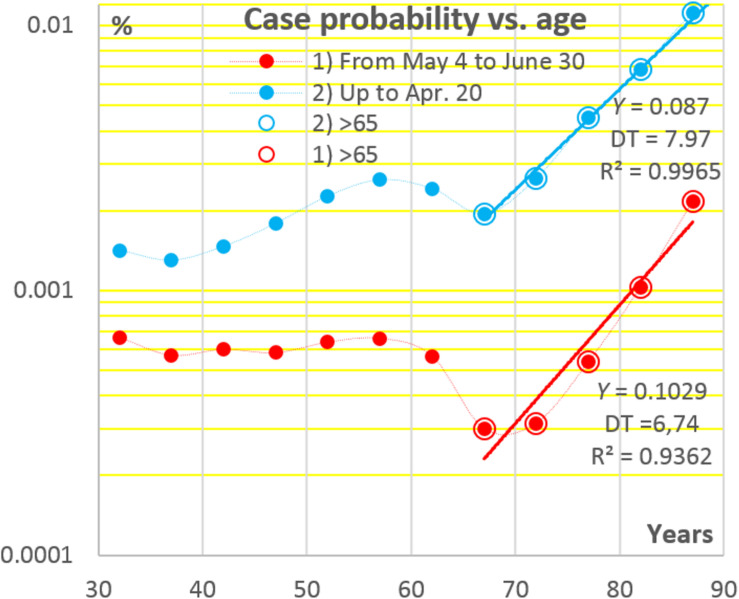
Age dependencies of the chances to become a registered COVID-19 patient in the Netherlands at two stages of the pandemic. Data sources: see Figure 1.

The aspect (iii) is illustrated in Figure 2 where data points have been constructed by dividing the numbers of registered COVID-19 cases in each of 5-years age intervals by population sizes in respective intervals. It may be seen that the chances to become a symptomatic COVID-19 patient change relatively little in the age interval from ca. 30 to 65 years, and then, at difference from seroprevalence, they rise exponentially, the DT being close to the sacramental 7 years. A possible reason of the sharp increases in the risk of becoming a symptomatic COVID-19 patient at an age above 65 may be that, in many West European countries, including the Netherlands, a substantial proportion of people at such ages find themselves concentrated in nursing houses (O’Driscoll et al., 2020).

*A priori*, the DT for COVID-19 CFR may differ from the general MRDT by any factor. However, all DTs fall within a strikingly narrow common range. This may lead to the somehow comforting conclusion that, in Nature, there does exist some order, which may be found even amidst anthropogenic mess, provided proper coordinates and viewpoint are chosen. This conclusion is at the same time disturbing as far as it relates to the gloomy phenomenon of aging.

Taken together, the above, on one hand, makes gerontology indispensable in dealing with COVID-19. On the other hand, the COVID-19 pandemic becomes a benchmark for gerontology to validate its concepts and advances.

## Grounds for Using Anti-Aging Interventions to Combat COVID-19

An important general issue in the crosstalk between physiology, gerontology, and epidemiology is the interpretation of the parameter γ of the Gompertz model (see section “Introduction”), which appears the same whether the model is applied to the general mortality or to COVID-19 CFR, as a measure of the population mean of the rate of decrease in the physiological capacities for resisting deadly impacts. Such impacts may originate not only from the outside of a body, but from the inside as well. The physiological functions that have evolved to control the inner forces that can produce such adverse events are no less essential for fitness and survival than the functions that evolved to control body interactions with the external conditions able to produce deadly damage (Golubev, 2009). In particular, the immune and blood coagulation systems are significant sources of potentially deadly internal impacts, which may be manifested as autoimmune conditions and intravascular blood coagulation events. Indeed, the incidences of autoimmune disorders and systemic septic responses, which are associated with intravascular coagulation, increase in the elderly (Vadasz et al., 2013; Mayr et al., 2014; Starr and Saito, 2014). The age-associated increases in the rates of exaggerated or biased immune responses to COVID-19, which are manifested as “cytokine storm” (Meftahi et al., 2020) and intravascular coagulation (Colantuoni et al., 2020), may result from that the ability of human body to control its responses to threats becomes increasingly compromised in the course of aging.

The age-dependent decrease in the abilities to control the endogenous pathogenic events and processes is manifested as the age-dependent increases in the incidences of the main non-communicable diseases, including cancers, cardiovascular conditions (such as atherosclerosis and heart failure), neurodegenerative diseases, and metabolic disturbances, such as obesity, metabolic syndrome, and type 2 diabetes. This list also includes several pulmonary pathologies, which are, notably, specific for the physiological system that is the most vulnerable to COVID-19. The conditions are the chronic obstructive pulmonary disease and the infectious pneumonias caused by several bacterial species. Importantly, the age-dependent increases in the incidences of the newly diagnosed cases of all these conditions and deaths attributed to them are close to exponential, and the respective DTs are close to MRDT (Newman and Easteal, 2017; Promislow, 2020; Santesmasses et al., 2020).

It thus comes out that there is nothing extraordinary in the relationships between aging and the rates of mortality associated with COVID-19. The relationships look as a particular manifestation of the age-dependent decreases in the physiological capacities for withstanding all sorts of deadly impacts. The resulting increases in mortality may be partly attenuated by acquired skills to avoid or beat off deadly impacts, in particular, by acquired immunity against infectious agents encountered earlier. Correspondingly, some infections may be tolerated better at later ages. However, this is not the case with newly emerged or rapidly mutating infectious agents such as SARS-CoV-2 or influenza viruses.

The age-dependent decline in the capacity of (human) body for withstanding all sorts of adverse impacts (the loss of the functional reserve) and the pathways of integration of innumerable molecular and cellular age-dependent changes into this decline are what the physiology of aging is about (Beek et al., 2016; Navaratnarajah and Jackson, 2017). An important open question is how comes that decreases in the functional reserves, which are usually close to linear, are translated into almost exponential increases in mortality? Different approaches to this issue are reviewed in (Golubev, 2009 and Golubev A., 2019; Golubev, 2019; Golubev and Anisimov, 2019).

Anyway, at least one of the aforementioned aging-associated pathologies, whose prevalence increases exponentially with age, preexist in up to 95% of COVID-19 victims, whereas those patients who have none of the conditions usually pass through the SARS-CoV-2 infection asymptomatically or experience only mild symptoms (Shamshirian et al., 2020; Zhang et al., 2020). It is then tempting to assume that the exponential increase in the prevalence of age-related pathology and the parallel increase in the deadly complications experienced by COVID-19 patients have a common background or are associated somehow otherwise. An immediate inference from this assumption is that the means that may help to prevent or treat the recently recognized COVID-19 may be found among the means suggested to mitigate the age-dependent decline in fitness and the associated development of long-known age-related pathological processes or even to slow down aging itself.

**FIGURE 3:**
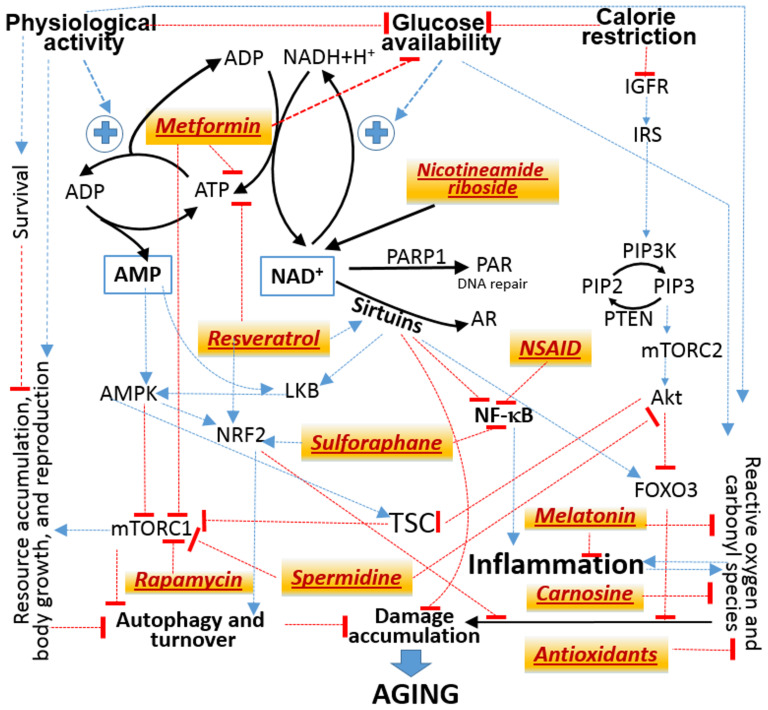
Attribution of the molecular targets of drugs suggested to treat both aging and COVID-19 (tan background) to signaling pathways from the availability of nutritional calories embodied in glucose to the physiological responses that allocate available resources between storage, growth and reproduction vs prevention and repair of molecular damage. Black solid lines designate mass transfer. Thin lines designate influences without specifying their mechanisms. Some may involve additional steps and intermediates, which are not shown. Blue arrows stand for activation or enhancement. Red bricks stand for inhibition or suppression. Abbreviations are explained in the main text.

The lists of substances that have been shown in at least some experiments with animal models, ranging from nematodes to primates, to extend their lifespans by presumably slowing down their aging may be found in the public databases Geroprotectors^3^ and DrugAge^4^, which have accumulated about four hundred entries. However, not more than a score of such substances are believed to produce consistent results in mammals and thus are evaluated for their effects in humans in clinical trials or/and population surveys (Qian and Liu, 2018; Piskovatska et al., 2019; Gonzalez-Freire et al., 2020). Those of the substances that have been suggested for treating COVID-19 are included in Figure 3. Remarkably, the molecular targets of each of them may be attributed to a few signaling pathways, which all converge to the molecular mechanisms that mediate the physiological responses to the two non-pharmacological interventions known to increase lifespan in all mammals studied so far in this regard, including humans. The interventions are: 1) diet calorie restriction and 2) physical activity promotion.

No single scheme can accommodate numerous important mechanistic details, which may be found in several reviews (Pan and Finkel, 2017; Vitale et al., 2019; Liu and Sabatini, 2020). Figure 3 is meant only to illustrate the fact that the molecular targets of many agents suggested to treat the adverse effects of both aging and COVID-19 may all be mapped to a common network of signaling pathways.

Another limitation of the present discussion is that only the substances that are commonly qualified as putative anti-aging agents (see the aforementioned databases and references) will be considered, whereas there are also other agents suggested to be useful in treating conditions associated with both aging and COVID-19. In particular, replenishing of vitamin D deficit, which is prevalent among the severe COVID-19 cases and the elderly, is suggested to mitigate the adverse effects of both aging (Hill et al., 2018) and COVID-19 (Daneshkhah et al., 2020; DeLuccia et al., 2020; Padhi et al., 2020). However, the biologically active endogenously hydroxylated derivatives of vitamin D are not known to target immediately the pathways shown in Figure 3 and are more likely to modulate the molecular machinery specific to the immune system (Sassi et al., 2018). The present discussion also does not touch upon the issue of how the pathways of SARS-CoV-2 interactions with its hosts, such as those related to the renin-angiotensin system (RAS) (Nishiga et al., 2020), change upon aging and may be directly modulated by agents and intervention suggested to correct the age-related abnormalities that result from these changes. This topic is discussed in several reviews, e.g., (Sahebnasagh et al., 2020; Quiles et al., 2020; Wang and Yang, 2020).

## COVID-19 vs Agents Suggested for Use Against Aging Based on the Stochastic Damage Theories

Approaches to understanding aging may be categorized into those that treat it either as resulting from the accumulation of stochastically damaged macromolecules, or as a programmed process.

The sources of the endogenous damage are reactive oxygen species (ROS), as it is implied in different versions of the free radical theory of aging (Harman, 1992; Barja, 2014), and the other byproducts of metabolism, such as reactive carbonyl species (Semchyshyn, 2014; Davies and Zhang, 2017), that are able to form stable covalent bonds with macromolecules, including proteins and DNA (Golubev et al., 2017a). Correspondingly, different antioxidants have long been examined for the ability to slow down aging and/or ameliorate age-related pathology. The pursuit of the first of the objectives proved to be largely futile (Aune et al., 2018; Jenkins et al., 2018). However, specific regimens of using antioxidants did show usefulness in therapy for and prevention of specific cardiovascular and neurologic conditions associated with aging. No wonder that oxidative stress has been implicated in COVID-19 complications, decreased antioxidant defenses are thought to contribute to the increased vulnerability of the elderly to COVID-19, and antioxidants have been suggested as adjuvant means against COVID-19 (Abouhashem et al., 2020; Delgado-Roche and Mesta, 2020; Nasi et al., 2020; Panfoli, 2020). Potent antioxidant activity may contribute to the ability of the indoleamine hormone melatonin, which has also been suggested as an adjuvant therapy for COVID-19 (Martín Giménez et al., 2020; Reiter et al., 2020), to ameliorate several adverse consequence of aging. The dipeptide carnosine, which is able to scavenge both ROS and reactive carbonyls (Hipkiss et al., 2016), has been suggested for treating COVID-19 in (Jindal et al., 2020).

The reasonability of enhancing the antioxidant defenses has prompted the suggestion that the activators of the transcription factor NRF2 (Figure 3) may be used to treat COVID-19 (Hassan et al., 2020; McCord et al., 2020). The physiological role of NRF2 is to enhance the expression of enzymes involved in protection against ROS-mediated damage and to stimulate the autophagy and turnover of damaged macromolecules upon increases in the amounts of reactive carbonyl-containing compounds, including metabolites oxidized by ROS. Among naturally occurring activators of NFR2, the isothiocyanate sulforaphane, which is derived from precursors abundant in cruciferous vegetables, has come into focus because of its reportedly anti-aging and anti-cancer activities (Santín-Márquez et al., 2019) and has been suggested as useful in COVID-19 treatment (Cuadrado et al., 2020).

Another agent able to stimulate autophagy is the polyamine spermidine. Its effects converge onto its ability to inhibit the protein complex mTORC1 directly and/or via inhibiting the kinase Akt, a component of the signaling pathway from insulin to mTORC1 via the protein complex TSC. Spermidine administration to rodents ameliorates age-associated pathology, including hypertension, and sperimidine-rich diets are associated with longevity in human populations (Madeo et al., 2018). Correspondingly, spermidine has been suggested as a component of therapy for COVID-19 (Gassen et al., 2020).

## COVID-19 vs Agents Suggested for Use Against Aging Based on the Theories of Programmed Aging

The involvement of mTORC1 in the prospective ability of putative anti-aging drugs to be useful in dealing with COVID-19 brings discussion to the alternative approach to aging, which treats it as programmed somehow.

The origin of this approach dates back to A. Weisman who envisioned the need for a special program that wouldterminate the lives of ancestors to spare space and resources for progeny. Later on, the natural reason for such a program was found in the elimination of frail or sick individuals from populations (Goldsmith, 2016; Muller, 2018). The conformity of these premises to the basics of the evolutionary theory is questionable (Kowald and Kirkwood, 2016).

Aging was also suggested to results from a program of increases in endocrine functions required for development and maturation. The increases were postulated to continue after optimum adult endocrine and metabolic conditions have been achieved and thus to drive the conditions out of the optimum (Dilman et al., 1986). If so, an appropriate retuning of the central neuroendocrine functions must be sufficient to slow down aging or even stop it.

A molecular biological derivative of this approach is the concept of cellular hyperfunction, specifically, of mTORC1, which is treated as a hub of the intracellular machinery whose activity enhances the anabolic processes needed to implement the program of growth and functional development during maturation. It has been hypothesized that the developmentally programmed high mTORC1 activity continues after the adult state has been achieved and pushes cellular and, by inference, body functions above their optimum levels. Thus, in order to slow down aging, it may be sufficient to inhibit mTORC1 (Blagosklonny, 2018).

Indeed, the antifungal substance rapamycin, whose mechanism of action is traced to mTORC1 inhibition, has been demonstrated to increase lifespan in mice under several experimental settings (Arriola Apelo et al., 2016; Blagosklonny, 2018). In humans, mTOR inhibition has been found to ameliorate some adverse consequences of aging (Blagosklonny, 2018; Lamming and Salmon, 2020), including low-grade chronic generalized inflammation nicknamed inflammaging, which has been implicated in COVID-19 pathogenesis (Fuellen et al., 2020). Based on the similarities between the cytokine storm upon COVID-19 and an exaggerated inflammatory response, rapamycin and its analogs have been suggested as adjuvant therapies for COVID-19 (Blagosklonny, 2020).

It does seem reasonable to associate inflammaging with proneness to exaggerated humoral immune responses manifested in the elderly as increases in the incidences of autoimmune disorders (Vadasz et al., 2013) and systemic septic responses (Mayr et al., 2014; Starr and Saito, 2014), sepsis being especially similar to the cytokine storm observed in severe COVID-19 cases (Colantuoni et al., 2020; Das et al., 2020; Li et al., 2020). According to the hyperfunction theory of aging in general, inflammaging is a particular manifestation of the primary aging-associated hyperactivity of almost everything in the body, including the immune system, predominantly its inborn and humoral branches, rather than a results of a reduced ability of its T-cellular branch to control B-cells, monocytes/macrophages and neutrophils. However, the primary factors of inflammaging may be traced to the age-associated hypofunction of the thymus (Thomas et al., 2020). Reduced levels of some leukocyte populations and respective interleukins may, through the dense network of regulatory feedbacks between the enormously numerous components of the cytokine and interleukin system, result in increased levels of some other interleukins, the general balance being biased toward predisposition to inflammatory responses.

To delineate feedback loops able to translate primary age-dependent decreases in the functions of defined leukocyte populations into secondary increases in the levels of interleukins implicated in the cytokine storm, one must wander through all possible interleukin-mediated pathways that control immune responses. This daunting task is far beyond the scope of the present discussion, whose main objective is limited to pinpointing the overlaps between the basic issues of COVID-19 research and gerontology. Simpler examples of aging-associated increases in functions because of compensatory responses to primary decreases in other functions or because of compromised negative feedbacks between different functions may be found in the neuroendocrine system, e.g., increases in the secretory activities of pituitary gonadotrophs because of decreases in the secretory capacities of their target glands (Golubev, 2009; Golubev et al., 2017a).

Noteworthy, another rationale (Terrazzano et al., 2020) for using mTOR inhibitors in therapy for COVID-19 is derived from the original field of their medical application, which is the prevention of allotransplant rejection by inhibiting immune responses against foreign antigens.

## Resveratrol, Metformin, and Physiological Resource Allocation

Resveratrol, whose place in Fig. 1 is close to the center, represents a large class of reportedly anti-aging naturally occurring compounds polyphenols (Russo et al., 2020), which have drawn attention as potentially useful in COVID-19 treatment (Annunziata et al., 2020; Quiles et al., 2020). Resveratrol administration to experimental organisms increases lifespan in several but not all experimental settings. Like other polyphenols, it can scavenge ROS, and the products of its oxidation upon ROS scavenging can activate NRF2. Resveratrol is also known to inhibit the activity of the transcription factor NF-κB, which is the central hub for integration of the stimuli that induce inflammation. However, the evidence that the non-steroidal anti-inflammatory drugs (NSAID), including aspirin, which can directly inhibit NF-κB, are useful against COVID-19 is controversial (Yousefifard et al., 2020), although aspirin is known to alleviate, in part by reducing inflammation, several age-associated pathologies, including some cancers and neurodegenerative diseases (Noreen et al., 2014; Ayyadevara et al., 2017).

Resveratrol can activate sirtuins (Kulkarni and Cantó, 2015; Bonkowski and Sinclair, 2016), which are involved in the repair of DNA and some forms of protein damage. These beneficial activities are associated with NAD^+^ consumption for the synthesis of poly(adenosyl ribose) (PAR) and adenosyl ribose, respectively. PAR is produced by the enzyme poly(adenosyl ribose) synthetase (PARP1). The ability of the sirtuin SIRT6 to activate PARP1 correlates positively with lifespan in different rodent species (Tian et al., 2019). However, because NAD^+^ depletion can result in a special form of regulated cell death called parthanatos, the known inhibitors rather than activators of PARP1 are suggested for COVID-19 treatment (Curtin et al., 2020).

The need to spare NAD^+^ for proper cell functioning suggests that the metabolic precursors of NAD^+^, such as nicotineamide riboside (NAR), may be used to combat aging by compensating for the aging-associated decrease in NAD^+^ availability (Bonkowski and Sinclair, 2016). NAD^+^ deficit has been reported to occur in cells upon SARS-CoV2 infection, and, accordingly, NAR has been suggested for COVID-19 treatment (Heer et al., 2020).

NAD^+^ is increasingly recognized as not only the coenzyme of dehydrogenases but also as the central component of the biochemical system implicated in sensing deficits of energy resources and transducing the respective signal to downstream effectors. Increases in NAD^+^ occur upon deficit in substrates whose oxidation in coupled with converting NAD^+^ into its reduced form NAD + H^+^ used in mitochondria for oxidative phosphorylation. A major source of such substrates is glucose.

NAD + H^+^ deficit reduces mitochondrial generation of ATP from ADP and, thus, increases the ADP/ATP ratio. Another cause of such increase is ATP hydrolysis to ADP coupled with generation of energy used in physiological functions. Increased energy demands are associated with increased generation of ADP. The first metabolic measure taken to maintain ATP availability is ADP transphosphorylation whereby two ADP molecules are converted into ATP and AMP. The resulting increase in AMP serves as another, besides NAD^+^, signal of energy deficit, in this case resulting from increased demands rather than from decreased supply. The AMP-activated protein kinase AMPK transduces the resulting signal to mTORC1 to inhibit it and thus to facilitate proteolysis as an extra source of energy substrates and to suppress anabolic processes involved in growth, storage and reproduction, which compete for resources with self-maintenance and repair.

NAD^+^ and AMP are recognized as the principal metabolic hubs through which the balance between the acquisition and consumption of resources is translated into the balance between their use for growth, storage and reproduction or for self-maintenance and, thus, for counteracting the inner forces that drive aging. Importantly, no resources may become available in the wild without physical activity required for different forms of foraging. Modern human lifestyle just uncouples resource acquisition from physical efforts, which have to be enforced with exercises in order to keep physiological balances at their optimum tuned in the course of evolution.

The ATP/ADP/AMP balance is where the antidiabetic biguanide metformin, another agent suggested to mitigate both aging (Anisimov, 2015; Campbell et al., 2017) and COVID-19 (Bramante et al., 2020; Menendez, 2020; Sharma et al., 2020) can operate. Metformin inhibits complex I of the mitochondrial electron transport chain and thus ATP production and in this way increases AMP up to a level mimicking the state of energy deficit resulting from increased energy demands. There are also data indicative of the ability of metformin to activate sirtuins (Cuyàs et al., 2018) and thus to mimic NAD^+^ deficit resulting from decreased supply of energy substrates. Moreover, metformin has been shown to inhibit mTORC1 in a way different from signaling pathways starting from AMP and NAD^+^. This effect is suggested to enhance the functions of T-cell subsets involved in the control of proinflammatory cytokines production (Bharath et al., 2020).

Without delving into mechanistic details, which are still not clear, it is possible to conclude from the above that the molecular targets of metformin are engaged in shifting the use of cell resources from storage, growth and reproduction to turnover, which is associated with elimination of damage, to counteracting the processes that make damage and to activating the mechanisms that repair damage.

## Pathophysiological Implications

At the physiological whole-body level, metformin improves glucose tolerance. Due to this effect, metformin is used to treat type II diabetes mellitus, which is associated with increased cells exposure to glucose. Notably, the risk of COVID-19 complications and associated deaths is increased upon reduced glucose tolerance even in non-diabetic patients (Drucker, 2020; Huang et al., 2020; Marhl et al., 2020; Zhu et al., 2020; Wang et al., 2020). Glucose tolerance decreases in the course of aging much because of decreases in skeletal muscle mass and functional capability (sarcopenia) and is partly responsible for increases in the incidences of aging-associated diseases, including even cancer (Golubev and Anisimov, 2019).

There are two aspects in the adverse effects of excess blood glucose. One is that glucose, being an energy source, shifts allocation of physiological resources away from mitigating the accumulation of endogenous damage (Figure 3). The other is that glucose itself produces damage because of the ability of any sugars and of methylglyoxal, a major byproduct of glucose metabolism via glycolysis, to form covalent adducts to the amino groups present in proteins and DNA (Sadowska-Bartosz and Bartosz, 2016). In the chemical terms, glucose is not unique in this regard. Other sugars, such as ribose, and many other endogenous molecules, such as catecholamines, are more reactive than glucose (Golubev, 1996; Golubev et al., 2017a). However, the ubiquitous presence and abundance of glucose make its role as a source of endogenous damage comparable with that attributed to ROS.

The relevance of the excessive chemical potencies of glucose and other metabolites to the theories of the evolutionary origin and development of aging is discussed elsewhere (Golubev, 2009; Golubev et al., 2017a, b; Golubev, 2019). As to COVID-19 pathophysiology, reduced glucose tolerance is a hallmark of poor metabolic fitness manifested in obesity and hyperlipidemia. Correlations between the metabolic and the immunological fitness are long recognized – see Golubev and Anisimov (2019) and references therein.

The direct pathogenic properties of glucose are especially significant for the vascular endothelium, which contacts blood glucose immediately. The endothelium not only prevents the direct contacts of blood constituents with the walls of the major blood vessels and, when it comes to microcirculation, with tissue cells, but also secretes numerous physiologically active substances, such as cytokines, eicosanoids, and nitric oxide, which can influence the conditions of blood and extravascular cells important for COVID-19 pathogenesis. Endothelial cells constitute the only type of resident cells that maintain their identity in all organs while being present in significant amounts there. In adult humans, the total mass of the endothelium is about 1 kg, and the endothelial surface is up to 7,000 m^2^ (Aird, 2013). Thus, the endothelium is a unique diffuse organ distributed throughout all other organs and tissues and capable of responding to stimuli in coherent ways despite all local peculiarities. This very organ is recognized as the primary target of SARS-CoV-2 attack and the responder to the attack. The ubiquity of changes caused by SARS-COV-2 in the endothelium contributes to the proneness of COVID-19 patients to multi-organ failure, as it is acknowledged in publications starting from Sardu et al. (2020) up to the latest (Froldi and Dorigo, 2020; Kaur et al., 2020).

With increasing age, the endothelial responses to any challenges, including COVID-19, become compromised making the whole body responses compromised. Indeed, any response of any tissue to any challenge must be supported with an adequate supply of oxygen, energy substrates, other relevant nutrients, and endocrine and paracrine factors. They all are delivered to effector cells by blood flow, which is regulated by the endothelium, and pass to the cells through the endothelium, many regulatory factors being produced in the endothelium. The claim that “one is as old as one’s endothelium” [Altschul (1954) cited in Triggle et al. (2020)] does not seem an overstatement.

The pathways and manifestations of aging in the endothelium have been discussed in numerous reviews, e.g., (Puca et al., 2012; Donato et al., 2015; Jia et al., 2019; Triggle et al., 2020). With all uncertainties and discordances, one consensus is that aging compromises the ability of the endothelium to respond to increased blood pressure and flow rate by increasing the production of nitric oxide (^⋅^NO). This deficiency is most evident in people with type II diabetes, obesity, metabolic syndrome, hypertension, and other conditions known to increase the risk of COVID-19 complications. Moreover, SARS-CoV-2 itself causes endothelial dysfunctions, including impaired ^⋅^NO production (Green, 2020).

Most publications addressing the relationships between COVID-19 and the endothelium focus on the RAS, which is directly implicated in SARS-CoV-2 infection, ^⋅^NO being largely ignored, although its interactions with RAS are known (Zhou et al., 2004). In the context of the involvement of the endothelium in the pathophysiological relationships between COVID-19 and aging, ^⋅^NO deserves more attention.

Being a potent vasodilator, ^⋅^NO also decreases platelet aggregation, mitigates inflammation, if not produced in excess (Sharma et al., 2007), and maintains the integrity of the endothelium, to name a few aspects of the ability of ^⋅^NO to counteract the age-associated changes that increase one’s vulnerability to COVID-19 complications. This makes the question “Are we as old as our NO?” (Sverdlov et al., 2014) quite relevant to the present discussion. In particular, Sverdlov et al. (2014) focused on the age-associated decrease in the sensitivity of platelets to the anti-aggregation effects of ^⋅^NO, thus highlighting another rationale, besides decreased ^⋅^NO production, for proposal to use ^⋅^NO donors or stimulants of ^⋅^NO production in therapy for COVID-19 (Williams et al., 2020; Yamasaki, 2020). Moreover, by nitrating the SH-groups present in the viral protease, ^⋅^NO can block SARS-CoV-2 entry into cells (Akaberi et al., 2020).

As to ^⋅^NO production, the abilities to ameliorate the age-associated functional impairments of the endothelium have been demonstrated for all agents featured in Figure 3: metformin (Triggle et al., 2020), resveratrol and other polyphenols (Oak et al., 2018; Li et al., 2019), NAR (Mateuszuk et al., 2020), sulforafan (Matsui et al., 2016), carnosine (Takahashi et al., 2009), antioxidants (Wray et al., 2012), spermidine (Ahrendt et al., 2020), melatonin (Rodella et al., 2013; Salmanoglu et al., 2016), and rapamycin, the effects of the latter being controversial (Reineke et al., 2015). This common property of such a diverse group of agents contributes to their common ability to ameliorate the consequences of aging, in particular, decreases in the functional reserves, including those mobilized in response to COVID-19. The reserves are obviously limited by the ability of the circulatory system, including microcirculation, to supply blood-borne energy substrates and regulatory factors to relevant cells and tissues.

Another common feature of these agents (see section “Grounds for Using Anti-Aging Interventions to Combat COVID-19”), is that the mechanisms of their action converge onto signaling pathways between the sensors of resource availability and the effectors of resource reallocation, which favor self-protection and maintenance upon a decrease in resource availability. However, each of the agents can reproduce only a part of body responses to the main determinants of resource availability, which are calorie acquisition with food and calorie spending by physical activity. Physical exercises and calorie restriction are known to improve endothelial conditions, including those that hinge on ^⋅^NO (Di Francescomarino et al., 2009; Korybalska et al., 2017; Otsuki et al., 2020), and such improvements are associated with the immunological benefits manifested in the reduction of inflammation and the enhancement of antiviral protection (Mohamed and Alawna, 2020).

Interestingly, according to the epidemiological data, the infectivity of SARS-CoV-2 depends on the age of an adult host much less than the pathogenic potential of the virus. The dependency of the probability of infection on age is almost flat in the range from ca. 20 to 70 years, as has been shown in India (Laxminarayan et al., 2020) and Spain (Pollán et al., 2000), whereas COVID-19 CFR is exponential throughout the entire adult age span (see section “Mortality Statistics as an Interface Between Gerontology and COVID-19 Research”). An analysis of epidemiological data in Spain, Italy and Japan (Omori et al., 2020) suggests that the age dependency of CRF is unrelated to the age dependency of the probability to contract SARS-CoV-2. Infection rate must depend primarily on the immunological factors, whereas death rate among those infected, on the pathophysiological factors.

A contribution of the age-dependent immunological changes, such as thymus involution (Kellogg and Equils, 2020) and immunosenescence (Brooke and Fahy, 2020) in combination with those caused by the endocrine and metabolic aging (Golubev and Anisimov, 2019) and endothelial dysfunctions (see above), to the age-dependent increase in COVID-19 CFR (sections “Introduction” and “Mortality Statistics as an Interface Between Gerontology and COVID-19 Research” herein) cannot be ignored. With that, when the epidemiological, physiological, and gerontological aspects of COVID-19 are considered together, the age-dependent changes in the endothelium appear at the forefront with regard to hospitalizations and deaths caused by COVID-19. Indeed, hospitalization rate secondary to COVID-19 has been found to correlate inversely with the maximal exercise capacity assessed up to 5 years earlier in study participants (Brawner et al., 2020). The conditions of the endothelium rather than of the immune system is what can contribute to limiting this capacity.

## Conclusion

All agents shown in Figure 3 are suggested as useful in therapies for COVID-19 based on the abilities to counteract aging and to decrease the risk of aging-associated diseases. However, each of these agents modifies only a part of signaling pathways initiated by decreased glucose availability, which may be achieved by both the restriction of dietary calories and the promotion of physical activity. Nevertheless, these agents are often qualified as calorie restriction mimetics (Shintani et al., 2018; Madeo et al., 2019). The development of the pharmacological mimetics of exercise and calorie restriction is claimed as promising for treating aging and preventing the development of aging-associated diseases, to which the recently emerged COVID-19 may be referred (Blagosklonny, 2020; Brooke and Fahy, 2020; Nir Barzilai et al., 2020).

The question however arises why prospective mimetics should be advised instead of the ready-to-use real things, whose effects are only partly reproduced by the mimetics? (Golubev, 2018). Indeed, calorie restriction is much more robust means of increasing the lifespan of experimental animals than any of the drugs believed to mimic it (Liang and Wang, 2018). Moreover, none of these drugs increases lifespan in experimental rodents by decelerating the rate of aging in terms of the Gompertz law (see sections “Introduction” and “Mortality Statistics as an Interface Between Gerontology and COVID-19 Research”), whereas calorie restriction does decelerate it (Golubev, 2009). Numerous epidemiological studies of human populations provide unequivocal evidence of the benefits of adequately balanced nutrition and physical activity for preventing age-associated diseases and mortality (Nieman and Wentz, 2019; Furrer and Handschin, 2020). It is increasingly recognized (da Silveira et al., 2020; Heffernan et al., 2020; Jakobsson et al., 2020; Neto et al., 2020) that lifestyle interventions, including promotion of physical activity, are the most reasonable, robust, and cost effective measures for reducing the burden of COVID-19 on public health.

The prospects for specific vaccines against SARS-CoV-2 and/or effective therapies for COVID-19 to be developed soon are currently uncertain. The time before they will become widely available may prove to be compatible with the time sufficient for one to experience an increase in the burden of aging, which exponentially increases the risk of death from COVID-19. The best thing one may do in such circumstances is to make use of interventions known to reduce most robustly the aging-associated decreases in fitness. The most convenient of such interventions are not the still underdeveloped regimens of using the anti-aging drugs according to the “geroscience” agenda (Nir Barzilai et al., 2020; Promislow, 2020), but the immediately available commencing to adequate physical and mental activities (Thune and Furberg, 2001; Gleeson et al., 2011; Lee et al., 2017; Celis-Morales et al., 2019; Kenyon, 2020; Neto et al., 2020) and involvement in social interactions (Yang et al., 2016; Kikusui, 2018; Banerjee et al., 2020; Santini et al., 2020). Physical exercises are clearly beneficial for endothelial functions, including those that hinge on the nitric oxide (see above) and RASs (Evangelista, 2020), as well as for immunity (Damiot et al., 2020). Specific regimens of exercises accounting for the peculiarities of the current epidemiological situation are being developed and tested (Dominski and Brandt, 2020; Ravalli and Musumeci, 2020; Zhu, 2020). “Metabolic health and lifestyle medicine should be a cornerstone of future pandemic preparedness” (Wood and Jóhannsson, 2020).

Unfortunately, the current anti-epidemic measures include social distancing associated with indoor dwelling, sedentary lifestyle, and relying on the digital mimetics of real life. Being justifiable as desperate attempts to reduce the overload of health care facilities at the onset of the pandemic, this is overtly at odds with enhancing the resistance to COVID-19 as well as to habitual aging-associated diseases in a long run.

The attempts to reduce the current spread of SARS-Cov-2 and the risk of COVID-19 are undertaken at the expense of increasing the future risks of this and the most prevalent aging-associated cardiovascular, neurological, and oncological conditions. For a proper balance to be found, gerontologists must be involved in crosstalk between virologists, (patho)physiologists, therapists, epidemiologists, and policy makers.

## Author’s Note

With regard to the sinificance of nitric oxide for relationships between aging and COVID-19, a recent paper published ahead of print provides an evidence of a strong cross-country correlation between the rates of deaths attributed to COVID-19 and the propotions of the NOS3 haplotype of the endothelial nitric oxide synthase (Guan et al., 2020).

## Data Availability Statement

The raw data supporting the conclusions of this article can be found in the following public database: https://www.mortality.org/cgi-bin/hmd/country.php?cntr=NLD&level=1.

## Author Contributions

The author confirms being the sole contributor of this work and has approved it for publication.

## Conflict of Interest

The authors declare that the research was conducted in the absence of any commercial or financial relationships that could be construed as a potential conflict of interest.

## Footnotes

1 https://pubmed.ncbi.nlm.nih.gov/?term=covid&sort=date

2 https://www.mortality.org

3 http://geroprotectors.org

4 https://genomics.senescence.info/drugs
